# Data in support of antioxidant activities of the non-extractable fraction of dried persimmon (*Diospyros kaki* Thunb.)

**DOI:** 10.1016/j.dib.2016.07.004

**Published:** 2016-07-12

**Authors:** Yoko Matsumura, Toshihiro Ito, Hisakazu Yano, Eiji Kita, Keiichi Mikasa, Masatoshi Okada, Azusa Furutani, Yuka Murono, Mitsuru Shibata, Yasue Nishii, Shin-ichi Kayano

**Affiliations:** a Department of Health and Nutrition, Faculty of Health Science, Kio University, 4-2-2 Umaminaka, Koryo-cho, Kitakatsuragi-gun, Nara 635-0832, Japan; b Department of Immunology, Nara Medical University, 840 Shijo-cho, Kashihara-city, Nara 634-8521, Japan; c Department of Microbiology and Infectious Diseases, Nara Medical University, 840 Shijo-cho, Kashihara-city, Nara 634-8521, Japan; d Nara City Hospital, 1-50-1 Higashikidera, Nara-City, Nara 630-8305, Japan; e Center for Infectious Diseases, Nara Medical University, 840 Shijo-cho, Kashihara-city, Nara 634-8521, Japan; f Department of Physical Therapy, Faculty of Health Science, Kio University, 4-2-2 Umaminaka, Koryo-cho, Kitakatsuragi-gun, Nara 635-0832, Japan

**Keywords:** Persimmon, Antioxidant activity, Oxygen radical absorbance capacity (ORAC), Model of the gastrointestinal tract

## Abstract

This data article is related to the research article entitled, “Antioxidant potential in non-extractable fraction of dried persimmon (*Diospyros kaki* Thunb.)” (Matsumura et al., 2016) [1]. We investigated antioxidant activities of the non-extractable fraction of dried persimmon fruits *in vitro* and *in vivo*. We evaluated both extracted fraction and non-extractable fraction, and reported that non-extractable fraction may possess significantly antioxidant potential *in vivo* on the basis of the oxygen radical absorbance capacity (ORAC). We showed our experimental raw data about antioxidant capacity of dried persimmon, plasma triglycerides (TG) and HDL-cholesterol (HDL-C), and this data article might contribute to evaluate real antioxidant capacity of other fruits and vegetables.

## Specifications Table

**Table:** 

Subject area	Biology
More specific subject area	Food Science
Type of data	Tables, figure
How data were acquired	Scavenging activities of the stable DPPH free radical were measured using a UV-visible spectrophotometer.
	ORAC values were measured using a microplate reader.
	TG and HDL-C level were measured using commercially available kits.
Data format	Raw
Experimental factors	The non-extractable fraction of dried persimmon was hydrolyzed for *in vitro* assay by heating at 90°C for 3 h with 5 mL of 1.2 N HCl–50% methanol solution, and was not hydrolyzed for *in vivo* assay.
Experimental features	The non-extractable fraction of dried persimmon showed antioxidant activity *in vivo*.
Data source location	Department of Health and Nutrition, Faculty of Health Science, Kio University, Nara, Japan
Data accessibility	Data is supplied in this article.

## Value of the data

• The non-extractable fraction of fruits and vegetables has been not regarded as subject of research. We evaluated antioxidant activities of the non-extractable fraction of persimmon *in vitro* and *in vivo* [1].

• These data are raw data obtained from our studies about the non-extractable fraction of dried persimmon. There are very few study of non-extractable fraction both *in vitro* and *in vivo*, and we disclose our raw data of antioxidant activities of non-extractable fraction of persimmon to the scientific researchers.

• The data in this article might contribute to evaluate antioxidant potential of other fruits and vegetables.

## 1 Data

*in vitro* antioxidant activities of each fraction of dried persimmon using both ORAC and DPPH radical-scavenging activities at three times was showed (Table 1, Table 2). Raw data of plasma ORAC value of each animal was showed in Table 3. Plasma concentration of TG and HDL-C were showed in Fig. 2. ORAC values of digestion mixtures of *in vitro* model of gastrointestinal digestion was showed in Table 4.

## 2. Experimental design, materials and methods

### 2.1 Sample preparation

**Fig. 1:**
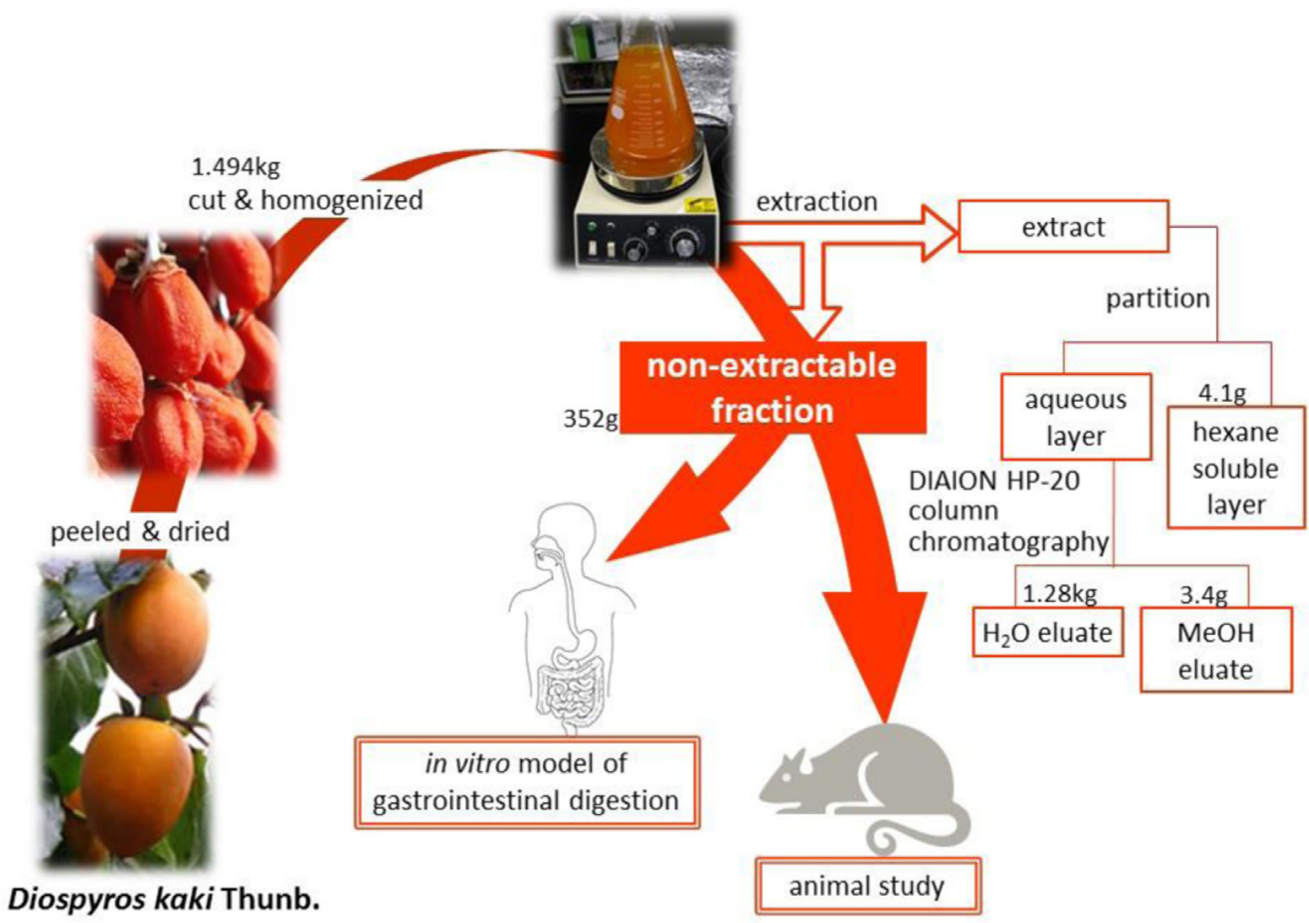
Sample preparation and flow of experiments.

**Fig. 2:**
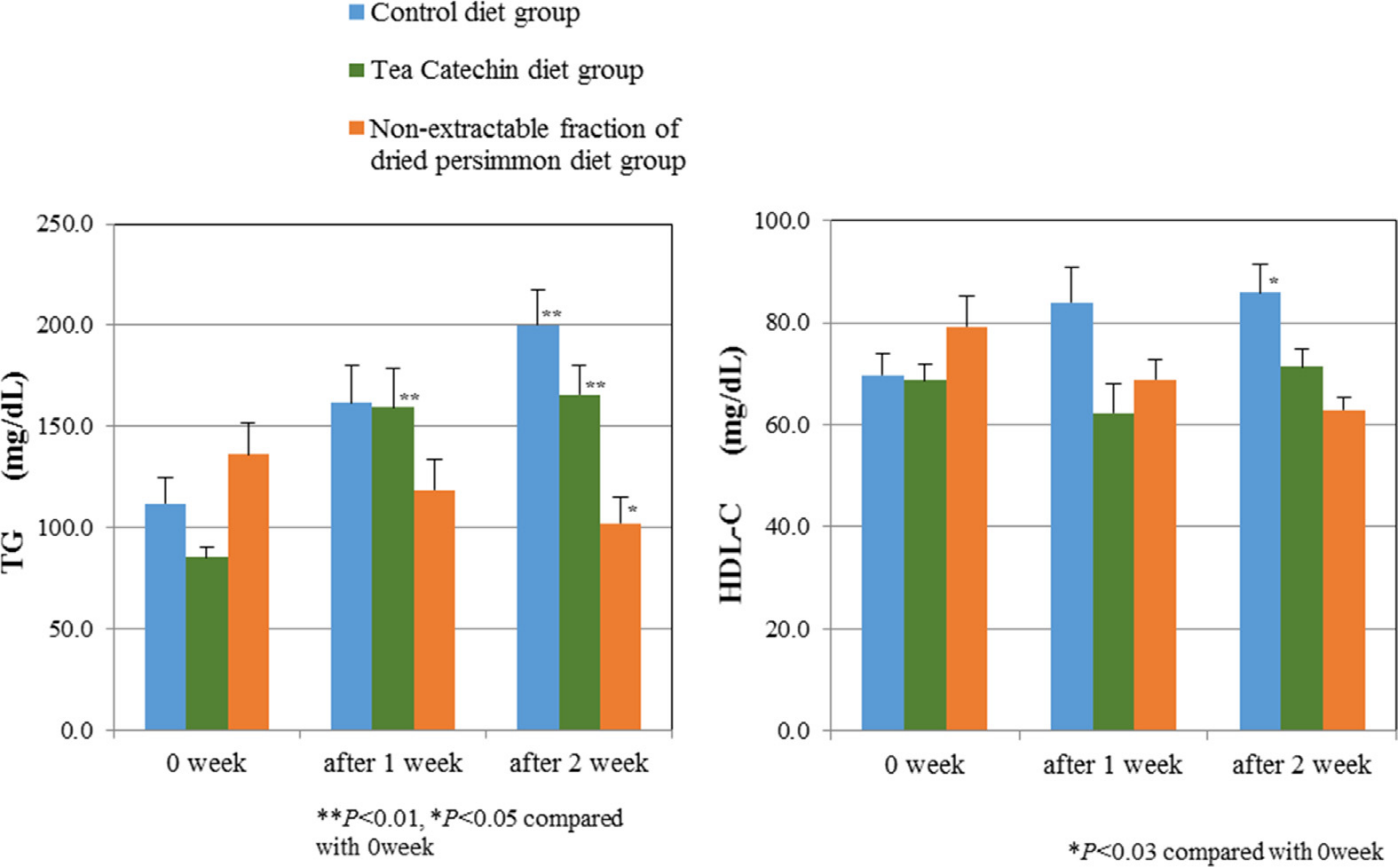
Plasma concentration of TG and HDL-C.

Dried persimmons (1.72 kg) were pitted (1.49 kg of edible portion), cut into small pieces, and homogenized in 10 L of 90% aqueous ethanol (EtOH), and the extract was filtered from the homogenate. Another 10 L of 90% aqueous EtOH was added to the residue. These extracting and filtering procedures were repeated three times and the combined filtrate was evaporated *in vacuo* to remove EtOH. The EtOH extract was dissolved in H_2_O and then added hexane, and was partitioned between H_2_O and hexane. The aqueous fraction was separated by column chromatography using a DIAION HP-20 gel with H_2_O as an eluting solution followed by elution with MeOH. All solutions were evaporated *in vacuo* to give hexane soluble layer (4.1 g), H_2_O (1.28 kg) and MeOH (3.4 g) eluates (Fig. 1). On the other hand, the extraction residue was dehydrated to obtain the non-extractable fraction as dried powder (352 g).

### 2.2 Preparation of hydrolyzed non-extractable fraction

Portions (100 mg) of the non-extractable fraction were heated at 90 °C for 3 h with 5 mL of 1.2 N HCl–50% MeOH solution in screw-capped tubes and were then centrifuged at 1750×*g* for 15 min at room temperature to obtain supernatants. Subsequently, 5 mL of 1.2 N HCl–50% MeOH solution was added to the precipitates and was then heated and centrifuged twice. Combined supernatants were diluted to 25 mL using MeOH.

### 2.3 Antioxidant activity of dried astringent persimmon

ORAC values of each fraction were measured according to a previously described method [2], [3] with slight modifications. This assay was performed based on the principle that antioxidant compounds delay decreases in fluorescein fluorescence following the addition of the peroxyl radical generator AAPH. ORAC assays were performed using an ARVOTM X4 microplate reader at an excitation wavelength of 485 nm and an emission wavelength of 535 nm. The fluorescence of each microplate well was recorded every 2 min over a 90-min period at 37 °C. The area under the fluorescence curve was calculated, and ORAC values for each sample were expressed as units for 1-μmol equivalents of Trolox. Each sample was measured in triplicate.

Antioxidant activities of extracts were estimated based on scavenging activities of the stable DPPH free radical using a previously described method [4] with slight modifications. Briefly, sample absorbance was measured at 517 nm using EtOH as the blank and ascorbic acid as the standard. Radical scavenging activities of samples are expressed as ascorbic acid equivalents.

The raw data at three times of ORAC values and DPPH radical-scavenging activities of dried persimmon fractions are shown in Table 1, Table 2.

### 2.4 Animal study

#### 2.4.1 Animal and feeding procedures

Eight-week-old male rats (Wistar strain) were purchased from Japan SLC (Hamamatsu, Shizuoka, Japan). Rats were randomly divided into three groups of eight animals, and each animal was individually housed. Rats of the control diet group were fed an AIN-93G-modified basal diet (CLEA Japan Inc., Tokyo, Japan) and those of the positive control group were fed tea catechin supplemented basal diet. A group of rats were fed a basal diet supplemented with 5% non-extractable fraction of dried persimmon instead of cellulose. Catechin contents in diets were calculated according to equivalent ORAC values in the non-extractable fraction diet, and β-cyclodextrin was added to reduce the bitterness of tea catechin.

Animals were fed *ad libitum*, and food intake and body weights were monitored daily for 3 weeks. Blood was collected from tail veins weekly and plasma was isolated and stored at −80 °C. All animal procedures were performed according to Kio University׳s guidelines for the care and use of laboratory animals, which are in compliance with the Japanese Law for the Humane Treatment and Management of Animals.

#### 2.4.2 Plasma ORAC assays

Plasma ORAC values were determined according to previously described methods [5]. Briefly, plasma samples were removed from storage at −80 °C and were slowly thawed and shaken using a vortex. Plasma aliquots (50 μL) were then transferred into microtubes and 100 μL of EtOH and 50 μL of H_2_O were added. Solutions were then shaken for 30 s using a vortex, and 200 μL of 0.75 M metaphosphoric acid was then added. Subsequently, mixtures were shaken using a vortex and centrifuged at 210×*g* for 5 min at 10 °C. Prior to ORAC analyses, 80 μL aliquots of supernatants were diluted into 420 μL of 75 mM phosphate buffer (pH 7.4) to obtain plasma solutions. Further dilutions (2–8 times) of plasma solutions were performed using 75 mM phosphate buffer (pH 7.4), and 20 μL aliquots were then transferred into microplates for ORAC assays.

The raw data of plasma ORAC values of 8 animals of each diet group are shown in Table 3.

#### 2.4.3 Plasma concentration of TG and HDL-C

Plasma concentration of TG was determined using commercially available kits (Triglyceride E-test WAKO, Wako Pure Chemical Industries, Osaka, Japan). HDL-C level were measured using commercially kits (HDL-cholesterol E-test WAKO, Wako Pure Chemical Industries, Osaka, Japan).

### 2.5 in vitro model of gastrointestinal digestion

#### 2.5.1 Conditions for in vitro digestion

The present model of gastrointestinal digestion was performed as previously described [6], [7], [8], [9]. The model describes a four-step procedure performed to mimic the digestive process in the oral cavity, stomach, small intestine, and large intestine at 37 °C.

Initially, 40 mg of α-amylase (20 units/mg, Wako Pure Chemical Industries, Osaka, Japan) in 0.1 M phosphate buffer (pH 6.9) containing 0.04% NaCl, and 0.004% CaCl_2_ was added to 300 mg samples of the non-extractable fraction and was incubated for 5 min for the oral digestion stage. Following oral digestion, gastric digestion was initiated by adjusting the pH to 2 using HCl and adding 60 mg of a porcine–pepsin solution (20 units/mg, Wako Pure Chemical Industries, Osaka, Japan) in 0.01 M HCl containing 0.9% NaCl. The mixture was then stirred gently for 1 h. Subsequently, 1 M NaHCO_3_ was added and gently stirred for 6 h with 35 mg of bile powder and 5 mg of pancreatin in 2 mL of 0.1 M phosphate buffer (pH 7.5) to adjust the pH to 6.5 and simulate small intestinal digestion. Finally, large intestinal digestion was simulated by fermentation of half amount of each samples following the addition of 14 mL of inoculum for 24 h under anaerobic conditions, and subsequent incubation in an anaerobic jar using an Anaero Pack system (Mitsubishi Gas Chemical Company, Inc., Tokyo, Japan). Inoculum was prepared by homogenizing feces from rats fed the control diet. Feces were collected under a flow of CO_2_ gas through the sampling bottle and were homogenized using a 10-fold prepared medium containing 2.5 g/L peptone, 2.5 g/L yeast extract, 0.9 g/L NaCl, 0.45 g/L KH_2_PO_4_, 0.027 g/L CaCl_2_·2H_2_O, 0.02 g/L MgCl_2_·6H_2_O, 0.008 g/L MnSO_4_·5H_2_O, 0.01 g/L CoCl_2_, 0.9 g/L (NH_4_)SO_4_, 0.008 g/L FeSO_4_·7H_2_O, 0.34 g/L K_2_HPO_4_, 0.003 g/L hemin, 0.001 g/L resazurin and 1.0 mL of Tween 80 in distilled water. As a control, crystalline cellulose was digested instead of the persimmon non-extractable fraction. And equal parts of fecal suspension was incubated for 24 h under anaerobic conditions.

#### 2.5.2 ORAC assays of digested samples

After each stage of the digestion process, samples were extracted with the same volume of MeOH using a stirrer and were then centrifuged at 1580×*g* for 5 min at room temperature. This procedure was repeated three times and supernatants were collected and evaporated *in vacuo* to obtain samples for ORAC assays.

The non-extractable fraction of dried persimmon was subjected to four processes of *in vitro* digestion and ORAC values were estimated for each stage at three times (Table 4).

## Figures and Tables

**Table 1:** ORAC value^a^ of dried astringent persimmon of three experiments.

	**hexane-soluble layer**		**H**_**2**_**O eluate**		**MeOH eluate**		**non-extractable fraction (hydrolyzed)**	
	units/g extract	units/100g edible portion	units/g extract	units/100g edible portion	units/g extract	units/100g edible portion	units/g	units/100g edible portion
1st	86	24	27	2267	2051	464	535	12608
2nd	131	36	25	2099	1839	416	536	12643
3rd	105	29	29	2449	2201	498	495	11669
mean	**107**	**30**	**27**	**2272**	**2030**	**459**	**522**	**12307**
S.D.	**22**	**6**	**2**	**175**	**182**	**41**	**23**	**553**

a ORAC values (units) are presented as units for 1 μmol of Trolox equivalent.

**Table 2:** DPPH radical-scavenging activities^a^ of dried astringent persimmon

	**hexane-soluble layer**		**H**_**2**_**O eluate**		**MeOH eluate**		**non-extractable fraction (hydrolyzed)**	
	units/g extract	units/100g edible portion	units/g extract	units/100g edible portion	units/g extract	units/100g edible portion	units/g	units/100g edible portion
1st	23.5	7	8.0	685	217	49	814	19195
2nd	25.6	7	8.7	746	183	41	828	19525
3rd	24.5	7	9.5	808	178	40	867	20445
mean	**24.5**	**7**	**8.7**	**746**	**193**	**44**	**836**	**19722**
S.D.	**1.0**	**0**	**0.7**	**61**	**21**	**5**	**27**	**648**

a DPPH radical scavenging activities (units) are expressed as units for 1 mg of ascorbic acid equivalent.

**Table 3:** Plasma ORAC value^a^ of animal study

	**Control diet group (units/mL)**			**Tea Catechin diet group (units/mL)**			**Non-extractable fraction of dried persimmon diet group (units/mL)**		
rat no.	0 week	after 1 week	after 2 week	0 week	after 1 week	after 2 week	0 week	after 1 week	after 2 week
No.1	1.3	1.7	2.1	1.9	1.3	1.7	0.9	1.7	3.8
No.2	1.7	1.7	1.7	1.4	1.6	2.9	1.8	2.3	1.8
No.3	1.7	2.4	2.2	1.4	1.2	3.0	2.0	4.2	2.8
No.4	1.5	2.1	2.5	1.3	1.4	2.6	1.5	3.0	2.4
No.5	1.4	2.1	2.3	1.8	1.7	3.0	2.2	2.5	3.1
No.6	1.6	1.2	1.3	1.6	2.2	2.3	1.0	3.1	N.D.
No.7	2.5	1.4	1.3	1.9	1.8	3.8	2.0	2.9	3.1
No.8	1.9	1.7	1.5	1.9	2.3	3.0	1.8	3.2	2.3
mean	**1.7**	**1.8**	**1.9**	**1.7**	**1.7**	**2.8**	**1.7**	**2.9**	**2.7**
S.E.	**0.14**	**0.14**	**0.17**	**0.09**	**0.14**	**0.21**	**0.16**	**0.26**	**0.25**

a ORAC values (units) are presented as units for 1 μmol of Trolox equivalent.

**Table 4:** ORAC values^a^ of digestion mixtures

	**oral cavity stage**		**stomach stage**		**small intestine stage**		**large intestine stage**		**fecal suspension**
	units/g of crystalline cellulose	units/g of non-extractable fraction of persimmon	units/g of crystalline cellulose	units/g of non-extractable fraction of persimmon	units/g of crystalline cellulose	units/g of non-extractable fraction of persimmon	units/g of crystalline cellulose	units/g of non-extractable fraction of persimmon	units/fecal equivalent
1st	1.1	15.6	32.1	77.9	112.3	90.2	472.4	742.9	589.2
2nd	1.2	18.5	44.9	60.6	100.5	82.3	457.5	673.6	602.2
3rd	1.7	18.7	88.0	69.4	105.1	90.0	551.9	872.0	536.5
mean	**1**	**18**	**55**	**69**	**106**	**88**	**494**	**763**	**576**
S.D.	**0**	**2**	**29**	**9**	**6**	**5**	**51**	**101**	**35**

a ORAC values (units) are presented as units for 1 μmol of Trolox equivalent.
